# The association between psychosocial factors, protective factors, and its associated triggers with psychological distress among Bolivian adolescents

**DOI:** 10.1038/s41598-023-39452-4

**Published:** 2023-08-03

**Authors:** Passakorn Suanrueang, Karl Peltzer, Zuchi Lkhamsuren, Lyen Krenz Yap

**Affiliations:** 1 Department of Health Education and Behavioral Sciences, Faculty of Public Health, Mahidol University, Ratchathewi, Bangkok Thailand; 2 Department of Psychology, College of Medical and Health Science, Asia University, Taichung, Taiwan

**Keywords:** Psychology, Signs and symptoms

## Abstract

The purpose of this study is to investigate the association between psychosocial factors, protective factors, and its associated triggers with psychological distress among Bolivian adolescents. This cross-sectional study was conducted by investigating the 2018 Bolivia global school-based student health survey (GSHS). The total number of students who participated in this survey was 7931, and the final sample was 7377. The mean age of the participants was 15.3 years (SD = 1.4). Psychological distress was assessed with a 2-item screener (loneliness and worry induced sleep disturbance). In all 22.3% of participants reported experiencing psychological distress, with 18.1% among adolescent males and 26.2% among adolescent females. In adjusted logistic regression analysis (AOR, 95% CI), there are two significant directions of association. One is the negative association, such as parental involvement as a protective factor. School adolescents who had more parental involvement were less likely to experience psychological distress. Parents understand problems or worries (0.64, 0.54–0.75, *p* < .001) and parents disregard privacy (0.69, 0.58–0.82, *p* < .001). On the other hand, many psycho-social factors are significantly positively associated with psychological distress. School adolescents who experience more psychosocial factors are more likely to experience psychological distress. Physical assault in the previous year (1.83, 1.59–2.11, *p* < .001), being bullied at school (1.27, 1.07–1.52, *p* < .01), being bullied outside of school (1.36, 1.15–1.61, *p* < .001), and being cyberbullied (1.60, 1.37–1.88, *p* < .001), were all significantly associated with psychological distress. Healthy relationships in a family, and interventions to reduce violence and bullying, should be encouraged and promoted.

## Introduction

Loneliness is one of the psychological consequences that affects people of all ages, including adolescents and school-age children^1^. Between 2012 and 2018, the global phenomenon of school loneliness increased in 36 out of 37 countries^2^. Loneliness is indicated as a mediator of anxiety, depression, and social expression^3^. Furthermore, loneliness, along with negative emotions, is a significant component of psychological distress that contributes to negative mental health outcomes, particularly depressive symptoms and suicidality^4^. Many studies have revealed the factors related to psychological distress. For example, a study using the 2015 Brazil National School Health Survey found that protective and risk factors for psychological health were the relationship with parents and the school context, including school-violent behaviors^5^. Loneliness was one of the aspects of psychological distress that was used to test as a psychological outcome in a group of adolescent-based studies in a global survey^6^. Loneliness has been reported to be a bond linked to anxiety and related symptoms that are emotional or affective circumstances^7^. The questions were used to be a latent variable of loneliness such as “How often you do feel lonely?” or “How many times have you been unable to sleep at night because of worry?”^6^. Moreover, the two questions were used as a screener for psychological distress among adolescents in different countries, with a prevalence of psychological distress of 18.6% in Caribbean nations^7^, 24.5% in Liberia^8^, 23.3% in Morocco^9^, 7.3% and 8.0% in Indonesia^10,11^, and 18.9% in Tonga^12^.

Sleep difficulty is an outcome of psychological distress, especially depressive symptoms, and anxiety. People who lack coping skills to deal with emotional difficulty are at increased risk of developing insomnia disorders^13^. Sleep disturbance was found in the general population including childhood and adolescence^14^. In a national survey of four adolescent nationalities (Argentinians, Chileans, Uruguayans, and Bolivians) who were injured by any means, in particular, physical attacks and being bullied, they were more prone to experiencing sleep problems because of the significant inducers of anxiety^15^.

The psychological health of adolescents is influenced by their positive or negative relationships with family members. In general, it is the responsibility of a family to settle the social standards of its members. The environment in which children and adolescents grow, especially in school life, influences their ways of dealing with life^5,16^. A family function is indicated to be a key component of improving healthy psychological health. This was found in a study of a cross-sectional study using national surveys of approximately 47,000 Norwegian adolescents^17^. Adolescents who are able to talk more with their parents have good mental health^17^. Additionally, parental involvement has been recognized as being a protective factor for adolescent psychological adjustment. Spending time with parents enhances adolescents’ sense of security^18^. Moreover, academic performance is either directly or indirectly linked to parental involvement in the schooling of adolescents^19^. The strong bond between parents and adolescents contributes to positive aspects that help protect against emotional and behavioural problems^20^. Adolescents with a high quality of parental involvement have better-coping skills to address stress, and it also reduces the risks of psychological distress such as depression and loneliness^21^. Furthermore, parental understanding and parental monitoring can positively contribute to adolescent mental well-being^22^. The evidence above mentions parental involvement as an essential aspect of monitoring the positive or negative psychological health of adolescents. The examples of parental involvement that have been used to investigate in the previous study, include parental supervision (checking to see if the homework of their children was done), parental connectedness (understanding the children’s problems and worries), and parental bonding (knowing what their children were doing with their free time)^6,9,11^.

On the other hand, there are significant risk factors for psychological distress. Physical assaults and fighting are major public health concerns among adolescents and school-age children worldwide. It is yet another psychological health trigger^23^. A cross-sectional study in a part of Ethiopia found about one-fourth and approximately 6% of 2424 adolescents experienced physical fights and physical attacks, respectively^23^. Additionally, the study conducted in Malaysia (4500 adolescents)^24^ and Pakistan (5177 school adolescents)^25^ found a connection between physical fighting and loneliness among adolescents. In Bolivia, a report from the Global School-based Student Health Survey conducted in 2012 indicated that nearly 37% of Bolivian students reported injuries, which included physical attacks and fights^15^. Additionally, they reported experiencing anxiety, which acted as triggers for insomnia. Moreover, similar findings were observed in other countries, including Argentina, Uruguay, and Chile^15^.

Adolescent males are more likely to experience twice the violence than adolescent females^23^. Similarly, a study based on the 2013 Global School-Based Health Survey in El Salvador found that compared to school-adolescent females, school-adolescent males were 3.55 times more at risk of physical fighting and had 2.16 times more experience with bullying^26^. Bullying in adolescents was found in over 37% of the results across national surveys of 40 countries, and it was found that school-adolescent boys experienced a wider range of bullying than girls, between 8.6 and 45.2%, while among girls, it was found from 4.8 to 35.8%^27,28^. In South Korea, one-tenth of 3000 study students aged 11–16 years were reported to have been bullied by their peers^29^. According to research from a nationwide survey of school adolescents in Indonesia, being the target of bullying three or more days a month is a trigger that increases psychological discomfort^30^. In Nepal, bullied adolescents were more likely to report mental health problems, including a higher risk of loneliness around 1.36 times, anxiety, and depression around 2.04 times^31^.

Bullying is a negative social action in which perpetrators behave toward victims directly through physical or verbal actions. It is also using social media platforms to bully others, which is called cyberbullying. Bullying on the online platform is also a significant cause of increasing psychological circumstances. Bullying victimization is undeniably the source of many psychological problems, especially anxiety and depressive symptoms^27^. Victimized adolescents who have experienced a high level of cyber victimization reported more loneliness and more difficult communication with parents^32^. A study among adolescents in Bolivia reported that there is a strong connection between cyberbullying victimization and the clinical presentation of psychological symptoms, especially depressive symptoms, and anxiousness^33^.

Using a conceptual framework from the World Health Organization (2005), we postulate that social environments among adolescents (social interaction with parents and peers), including parental involvement, peer support and psychosocial factors (interpersonal violence and bullying victimization) are associated with psychological distress^11^. However, studies evaluating the association between protective, psychosocial factors and psychological distress are lacking in Bolivia. The evaluation of protective, psychosocial factors and psychological distress in Bolivia represents a crucial addition to the literature because adolescents in Bolivia encounter various adverse problems^15,33^. In order to understand the positive and negative factors in order to establish a plan to prevent and control psychological distress in teenage populations, the study aimed to examine the protective aspects and risk factors that are associated with psychological distress among Bolivian adolescents.

## Methods

### Source of data

This cross-sectional study was conducted by investigating the 2018 Bolivia Global School-Based Student Health Survey (GSHS), which was a school-based survey of students between 2nd Secondary and 6th Secondary grades in Bolivia. Participants were selected randomly through a two-step process. The first step involved selecting schools, and the second step involved selecting students aged 11–18 years from the classes within those schools. The inclusion criteria were all students present in the selected classrooms regardless of age. The students subsequently provided their answers to each question on a computer-readable answer sheet by themselves. The study was carried out using the publicly available data recorded on the World Health Organization (WHO) website. The total number of students who participated in this survey was 7931 (total response rate was close to 80%)^34^. Of the total sample of 7931, 564 had missing values on the outcome variable (psychological distress), which were removed resulting in our study sample of 7377.

### Measures

The GSHS measurement is similar to the CDC Youth Risk Behavior Survey^35^, with test and re-test reliability known. Furthermore, the GSHS showed that “the average agreement between the test and the re-test is 77%, and the Cohen kappa is 0.47”^36^. This study used the Bolivia GSHS questionnaire^34^ that is consisted of three core modules, including (1) psychological distress (loneliness, sleep disturbances, anxiousness); (2) protective factors (the relationship with parents and having close friends), and (3) psychosocial factors (interpersonal violence, being bullied).

### Outcome variable

Psychological distress variables were measured with two items, including (1) “During the past 12 months, how often have you felt lonely?” and (2) “During the past 12 months, how often have you been so worried about something that you could not sleep at night?” Response options were “Never = 1, Rarely = 2, Sometimes = 3, Most of the time = 4, Always = 5”, and were coded as “Never = 0, Rarely = 1, Sometimes = 1, Most of the time = 2, Always = 3.” Scores of the two items were summed, and scores of three or more were defined as psychological distress. Coding and scoring of this 2-item psychological distress measure followed previous studies^9,10^. Cronbach alpha for the 2-item psychological distress measure was 0.61. The inter-item correlation was 0.44.

### Co-variables

*Sociodemographic factors* included sex and age group.

*Protective factors* included four items on parental involvement and one item on having close friends. The four parental involvement items are the following: (1) parents check homework: “During the past 30 days, how often did your parents or guardians check to see if your homework was done?” (2) parents understood problems and worries: “During the past 30 days, how often did your parents or guardians understand your problems and worries?” (3) parents know what you were doing with your free time: “During the past 30 days, how often did your parents or guardians really know what you were doing with your free time?” (4) parents go through your things without your approval: “During the past 30 days, how often did your parents or guardians go through your things without your approval?” Response options were “Never = 1, Rarely = 2, Sometimes = 3, Most of the time = 4, Always = 5”. Items 1–3 were coded most of the time or always = 1 and never, rarely, or sometimes = 0, and item 4 never or rarely = 1 and sometimes, most of the times or always = 0.

Having close friends. (“How many close friends do you have?”) (number).

*Psycho-social factors* included the number of students experiencing three components:

1. interpersonal violence included two items; physically attacked (“During the past 12 months, how many times were you physically attacked?” with coded 0 = 0 times, 1 = 1–12 or more times) and physical fighting in the past 12 months (“During the past 12 months, how many times were you in a physical fight?” with coded 0 = 0 times, 1 = 1–12 or more times)
2. being bullied included three items (response in yes, no questions); being bullied at school (“During the past 12 months, have you ever been bullied on school property?”), being bullied outside school (“During the past 12 months, have you ever been bullied when you were not on school property?”), and being cyberbullied in the last 12 months (“During the past 12 months, have you ever been cyberbullied?”).

### Data analysis

Descriptive statistics were performed using frequencies to describe categorical variables. Testing differences in proportions were calculated using Pearson's chi-square tests. Logistic regression was utilized to present the effects measured among variables by presenting crude and adjusted odds ratios, and 95% confidence intervals. Missing values were not included in the analysis. The statistical software that we used in this study is Stata version 16.0 (Stata Corporation, College Station, TX, USA), taking the multi-stage sampling and weighting of the data into account.

### Ethical considerations

The study protocol was approved by the Ministry of Health, Bolivia, and a national ethics committee. The necessary approvals and permits, including informed consent, were obtained from the participating schools, the parents, and the students before the survey was administered. This study used publicly available data from the website of the World Health Organization (WHO), which contains anonymous individual information; hence institutional review board approval was not obtained. All methods were carried out in accordance with relevant guidelines and regulations.

## Results

### Sample and psychological distress characteristics among adolescents in Bolivia

The sample consisted of 7931 Bolivian adolescents. After missing data of the outcome variable were removed, the sample included 7377, with approximately 22.3% showing psychological distress. Adolescent females had a higher percentage of psychological distress, accounting for 26.2%, whereas adolescent males accounted for approximately 18.1%. The number of adolescents experiencing psychological distress was found to increase with age after 15 (Table 1).

**Table 1:** Sample and psychological distress characteristics among school adolescents in Bolivia, 2018 (% are weighted).

Variable	Missingn (%)	Samplen (%)	Psychological Distress n (%)						
			Both n (%)	Male n (%)	χ^2^ (df)	*p*	Female n (%)	χ^2^ (df)	*p*
All (n = 7931)	554 (7.0)	7377 (100.0)	1634 (22.3)						
Sociodemographic									
Gender	685 (8.6)	7246 (100.0)							
Male		3742 (50.7)	679 (18.1)						
Female		3504 (49.3)	916 (26.2)						
Age group in years	727 (9.2)	7293 (100.0)							
≤ 11–14		2196 (30.4)	446 (20.5)	180 (16.3)	1.69 (3)	0.183	252 (24.0)	3.71 (3)	0.019
15		1479 (20.2)	310 (20.7)	136 (18.3)			171 (22.9)		
16		1500 (20.1)	345 (23.4)	134 (17.7)			205 (29.1)		
17 +		2118 (29.2)	510 (24.3)	222 (20.0)			284 (29.1)		
Protective factors									
Parents check homework (mostly or always)	902 (11.4)	2275 (31.9)	423 (18.5)	195 (17.3)	0.57 (1)	0.453	219 (19.4)	39.02 (1)	***
Parents understood problems/worries (mostly or always)	810 (10.2)	2191 (30.2)	353 (16.2)	156 (13.6)	14.47 (1)	***	185 (18.2)	37.28 (1)	***
Parents know what you were doing with your free time (mostly or always)	888 (11.2)	2715 (37.4)	528 (19.6)	214 (16.1)	5.72 (1)	0.021	298 (22.4)	10.60 (1)	0.002
Parents go through your things without your approval (never or rarely)	954 (12.0)	5524 (77.7)	1124 (20.4)	482 (17.2)	6.74 (1)	0.013	622 (23.8)	15.93 (1)	***
Number of close friends	802 (10.1)	7257 (100.0)							
0		752 (10.7)	281 (37.1)	115 (30.2)	17.27 (3)	***	156 (45.1)	34.53	***
1		1049 (14.7)	282 (27.2)	96 (20.6)			181 (32.7)		
2		1279 (17.8)	302 (23.2)	122 (20.3)			171 (25.4)		
3 or more		4177 (58.8)	733 (17.7)	326 (14.7)			394 (20.9)		
Psycho-social factors									
Interpersonal violence									
Physically attacked past 12 months	721 (9.1)	2038 (28.1)	668 (33.4)	298 (28.0)	137.40 (1)	***	346 (38.9)	105.05 (1)	***
Physical fighting	726 (9.2)	2190 (30.1)	570 (26.2)	315 (21.2)	15.87 (1)	***	234 (36.0)	40.51 (1)	***
Being bullied									
Being bullied at school	1011 (12.8)	1734 (24.8)	536 (31.2)	217 (24.9)	35.58 (1)	***	310 (37.5)	52.31 (1)	***
Being bullied outside of school	850 (10.7)	1547 (21.6)	497 (32.2)	190 (24.5)	36.59 (1)	***	297 (40.2)	62.66 (1)	***
Being cyberbullied in the past 12 months	886 (11.2)	1544 (21.5)	517 (34.1)	182 (27.2)	36.59 (1)	***	325 (39.6)	70.37 (1)	***

***p < .001.

In four dimensions of protective factors related to the relationship with parents, the percentage of adolescents experiencing psychological distresses was found to be a lower percentage than one-third in three dimensions for both genders. These dimensions were: parents check homework (18.5%), parents understood problems and worries (16.2%), and parents know what you were doing with your free time (19.6%). When these dimensions were analysed separately by gender, it was found that these three dimensions of parental involvement were associated with a lower percentage (less than 23%) of adolescents experiencing psychological distress (Table 1).

On the other hand, among both gender of Bolivian adolescents, in the cases of adolescents experiencing physically attacked in the past 12 months, were found a higher number experienced psychological distress approximately 40%, and physical fighting was around 35%. There were victimized adolescents of bullying who suffered from psychological distress approximately one-third. Being cyberbullied in the past 12 months (34.1%), was the majority of victimization of bullying experiencing psychological distress. Followed by being bullied outside school (32.2%) and being bullied at school (31.2%). The percentage of adolescents experiencing psychological distress was found to decrease with the number of close friends (Table 1).

### Associations with psychological distress among adolescents in Bolivia

The crude and adjusted odds ratios with a 95% confidence interval are presented in Table 2, showing the association between sociodemographic factors, protective factors, psycho-social factors, and psychological factors with psychological distress by gender.

**Table 2:** Logistic regression analysis of factors associated with psychological distress among adolescents in Bolivia.

Variables	Crude Odds Ratio (COR) (95% CI)			Adjusted Odds Ratio (AOR) (95% CI)^a^		
	Both	Male	Female	Both	Male	Female
Sociodemographic						
Gender						
Male	1 (Reference)			1 (Reference)		
Female	0.62 (0.55–0.71)***			0.60 (0.52–0.71)***		
Age group in years						
Age 11 to 14	1 (Reference)	1 (Reference)	1 (Reference)	1 (Reference)	1 (Reference)	1 (Reference)
Age 15	1.01 (0.87–1.18)	1.15 (0.91–1.45)	0.94 (0.76–1.17)	1.06 (0.89–1.25)	1.25 (0.96–1.62)	0.94 (0.72–1.22)
Age 16	1.19 (0.98–1.44)	1.10 (0.84–1.46)	1.31 (1.03–1.65)*	1.27 (1.02–1.58)*	1.23 (0.89–1.69)	1.30 (1.01–1.68)*
Age 17 and over	1.24 (1.07–1.45)*	1.29 (1.01–1.64)*	1.30 (1.04–1.63)*	1.35 (1.13–1.62)**	1.43 (1.13–1.82)***	1.31 (1.03–1.66)*
Protective factors						
Parental involvement						
Parents check homework	0.73 (0.63–0.84)***	0.93 (0.76–1.13)	0.58 (0.48–0.69)***	0.89 (0.79–1.01)	1.07 (0.91–1.27)	0.78 (0.64–0.94)*
Parents understood problems and worries	0.59 (0.50–0.68)***	0.62 (0.49–0.80)***	0.53 (0.43–0.66)***	0.64 (0.54–0.75)***	0.64 (0.47–0.87)*	0.64 (0.52–0.80)***
Parents know what you were doing with your free time	0.78 (0.67–0.91)***	0.79 (0.65–0.96)*	0.73 (0.60–0.89)***	1.08 (0.90–1.31)	1.01 (0.78–1.31)	1.15 (0.93–1.43)
Parents go through your things without your approval	0.66 (0.56–0.77)***	0.75 (0.60–0.94)*	0.62 (0.49–0.79)***	0.69 (0.58–0.82)***	0.75 (0.59–0.94)*	0.65 (0.50–0.85)***
Having close friends						
Having close friends (1 or more)	0.73 (0.69–0.77)***	0.75 (0.71–0.81)***	0.70 (0.65–0.76)***	0.74 (0.68–0.78)**	0.73 (0.70–0.77)***	0.72 (0.69–0.77)***
Psycho-social factors						
Interpersonal violence						
Physically attacked past 12 months	2.30 (2.05–2.58)***	2.37 (2.04–2.76)***	2.30 (1.95–2.71)***	1.83 (1.59–2.11)***	1.93 (1.58–2.36)***	1.74 (1.44–2.09)***
Physical fighting	1.37 (1.23–1.53)***	1.40 (1.18–1.67)***	1.79 (1.48–2.15)***	1.08 (0.92–1.26)	0.99 (0.77–1.26)	1.20 (0.94–1.52)
Being bullied						
Being bullied at school	1.92 (1.67–2.21)***	1.79 (1.47–2.18)***	2.09 (1.70–2.57)***	1.27 (1.07–1.52)**	1.20 (0.93–1.54)	1.33 (1.00–1.78)*
Being bullied outside of school	2.02 (1.77–2.31)***	1.74 (1.45–2.10)***	2.37 (1.90–2.96)***	1.36 (1.15–1.61)***	1.19 (0.95–1.49)	1.51 (1.11–2.06)*
Being cyberbullied past 12 months	2.29 (1.98–2.66)***	2.05 (1.69–2.49)***	2.38 (1.92–2.94)***	1.60 (1.37–1.88)***	1.61 (1.27–2.06)***	1.60 (1.28–2.00)***

An adjusted for all variables in the table; ***p < .001, **p < .01, *p < .05.

About sociodemographic factors, compared to adolescent males, females were less likely to have psychological distress (COR = 0.62; 95% CI = 0.55–0.71, *p* < 0.001, AOR = 0.60; 95% CI = 0.52–0.71, *p* < 0.001). The prevalence of psychological distress increases with age. Compared to other aged groups, older adolescents aged 17 years and over were more likely to have psychological distress (AOR = 1.35; 95% CI = 1.13–1.62). Male to female: AOR = 1.43; 95% CI = 1.13–1.82, *p* < 0.001: AOR = 1.31; 95% CI = 1.03–1.66, *p* < 0.05 (Table 2).

Regarding protective and risk factors, all items of protective and risk factors for psychological distress were significantly associated with psychological distress in crude odds ratios. However, after the OR was adjusted, some items of these factors were strongly significantly associated, such as (1) parents understood problems and worries (AOR = 0.64; 95% CI = 0.54–0.75, *p* < 0.001), male: female: AOR = 0.64; 95% CI = 0.47–0.87, *p* < 0.05: AOR = 0.64; 95% CI = 0.52–0.80, *p* < 0.001. (2) parents go through your things without your approval (AOR = 0.69; 95% CI = 0.58–0.82, *p* < 0.001), male: female: AOR = 0.75; 95% CI = 0.59–0.94), *p* < 0.05: AOR = 0.65; 95% CI = 0.50–0.85, *p* < 0.001 (Table 2).

Regarding psycho-social factors, physically attacked in the past 12 months and physical fighting were significantly associated with psychological distress in COR in both sexes, physically attacked: physical fighting: (COR = 2.30; 95% CI = 2.05–2.58, *p* < 0.001: COR = 1.37; 95% CI = 1.23–1.53, *p* < 0.001. However, after the OR was adjusted, physically attacked was the only factor that was a significant risk factor for psychological distress in both genders. (AOR = 1.83; 95% CI = 1.59–2.11, *p* < 0.001). Male: female: AOR = 1.93; 95% CI = 1.58–2.36, *p* < 0.001: AOR = 1.74; 95% CI = 1.44–2.09, *p* < 0.001. (Table 2).

Regarding psychological factors, all three psychological factors, including being bullied in school, being bullied outside school, and being cyberbullied past 12 months, were strongly significantly associated with psychological distress in COR and AOR in both genders. Being bullied at school (COR = 1.92; 95% CI = 1.67–2.21, *p* < 0.001: AOR = 1.27; 95% CI = 1.07–1.52, *p* < 0.01), being bullied outside the school (COR = 2.02; 95% CI = 1.77–2.31, *p* < 0.001: AOR = 1.36; 95% CI = 1.15–1.61, *p* < 0.001), and being cyberbullied in the past 12 months (COR = 2.29; 95% CI = 1.98–2.66, *p* < 0.001: AOR = 1.60; 95% CI = 1.37–1.88, *p* < 0.001). After the OR was adjusted, being cyberbullied was the only factor that was a strongly significant risk factor (*p* < 0.001) for psychological distress in both genders. Male: female: AOR = 1.61; 95% CI = 1.27–2.06: AOR = 1.60; 95% CI = 1.28–2.00 (Table 2).

**Figure 1:**
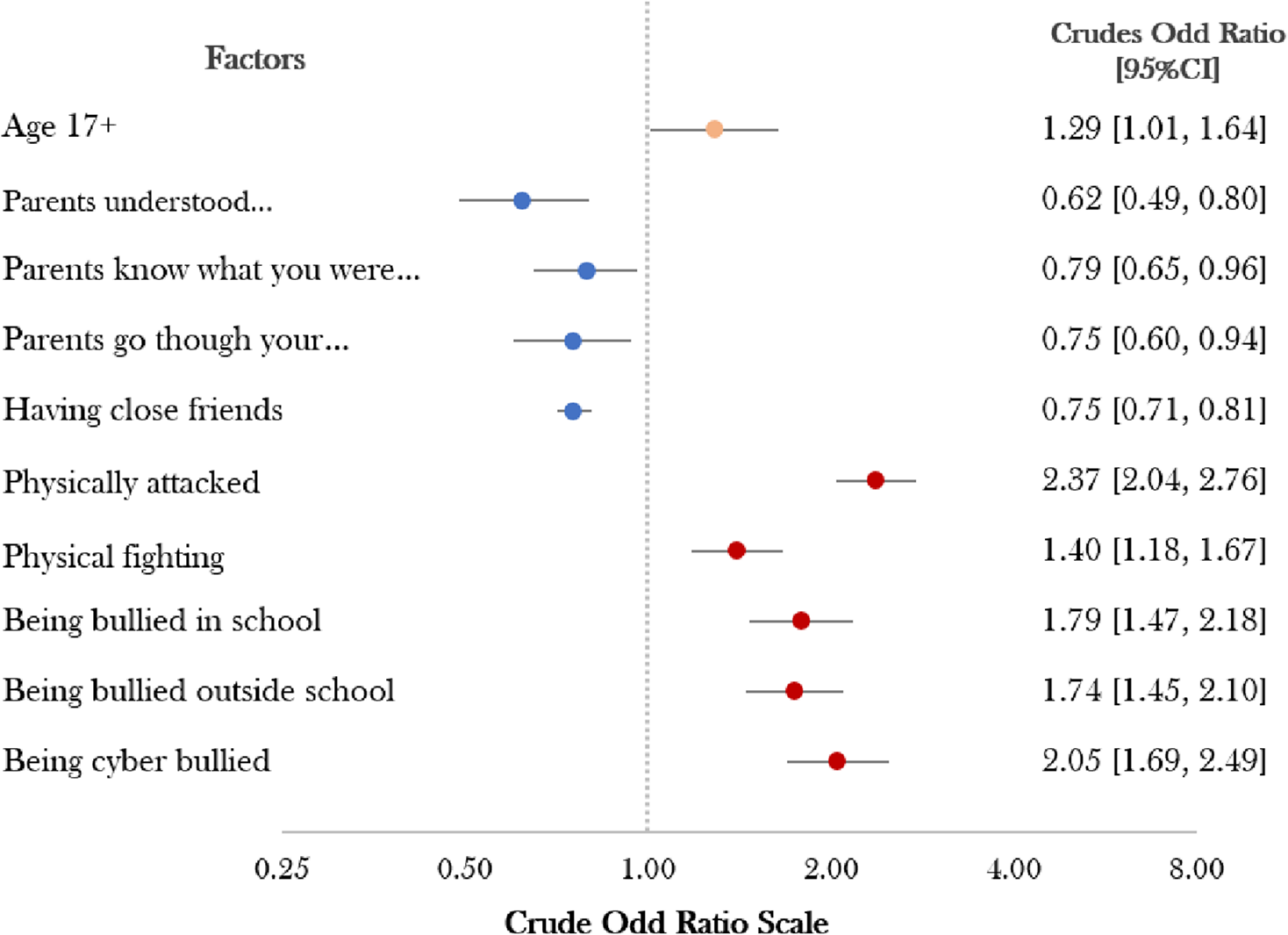
The crude odd ratio for protective and psychosocial factors in adolescent males.

Among Bolivian adolescent males, physical fighting and being cyberbullied were found to be the most significant predictors of psychological distress, with crude odds ratios of 2.37 and 2.05. Adolescent males aged 17 and over were found to be particularly vulnerable to psychological distress compared to other age groups. Contrary to the previously mentioned risk factors, parental involvement and having close friends were found to be protective against psychological distress in this study, with crude odds ratios of less than 1 (Fig. 1).

**Figure 2:**
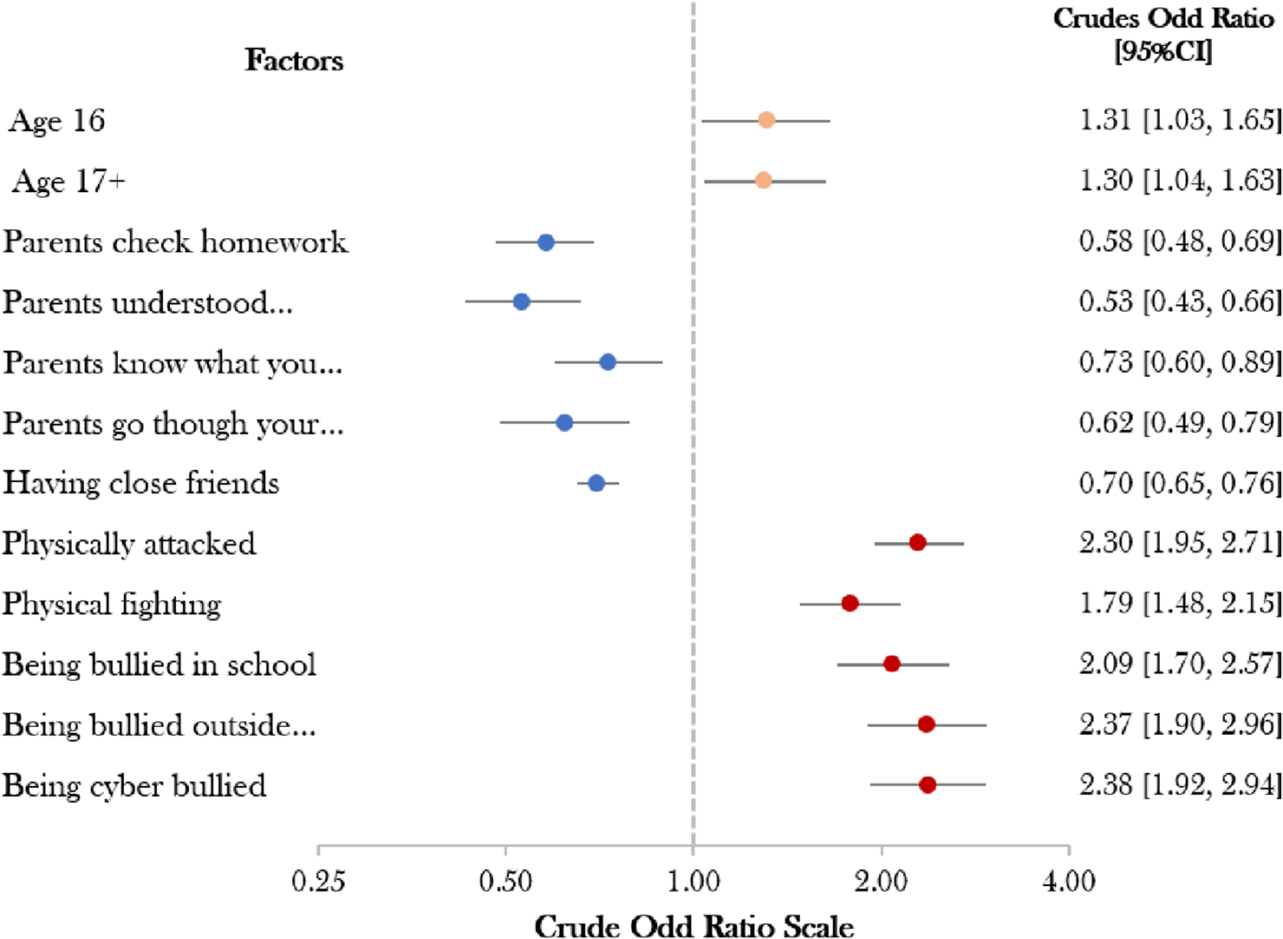
The crude odd ratio for protective and psychosocial factors in adolescent females.

Cyberbullied, bullied outside school, and bullied at school were found to be significant predictors of psychological distress among Bolivian adolescent females, with crude odds ratios of 2.38, 2.37, and 2.09, respectively. The physical attack was also discovered to be a predictor of psychological distress in adolescent females. Adolescent females aged 16 and older were also identified as being at higher risk of psychological distress compared to other age groups. In contrast, similar to adolescent males, parental involvement and having close friends were found to be protective against psychological distress, with crude odds ratios of less than 1 (Fig. 2).

## Discussion

This recent study aimed to examine the associations between protective and psychosocial factors and psychological distress among Bolivian adolescents. We found a high prevalence of psychological distress (22.3%), similar to in Morocco (23.3%)^9^ and in Liberia (24.5%)^8^, and higher than in Tonga (18.9%)^12^, in Caribbean nations (18.6%)^7^, and in Indonesia (7.3–8.0%) in Indonesia^10,11^, calling for strategies to prevent and control psychological distress among adolescents in Bolivia.

In this study interpersonal violence (physically attacked, and physical fighting) and being bullied (being bullied online, at and outside schools) are the main predictors of contributing to psychological distress (loneliness and worry-induced sleep disturbance) among Bolivian adolescents. These current findings are consistent with those of previous studies, including a study in schools in countries of the Western Pacific islands^37^, a study of adolescents in four Caribbean countries^38^, and a study of Indonesian female adolescents^10^, which all found that violence was associated with loneliness. There is a study indicating the circumstances relation between violence and loneliness. It is indicated that factors such as the classroom environment and experiences of victimization can contribute to feelings of loneliness, which in turn can increase the likelihood of engaging in overt and relational violence^39^. Furthermore, some research indicated that violence and being bullied can have the power of stimulating sleep problems, which is one of the branches of psychological distress^40,41^. Violence can have a significant impact on sleep patterns and overall sleep quality. Exposure to violence, whether as a witness or a victim, can lead to sleep disturbances such as difficulty falling asleep, difficulty staying asleep, and frequent nightmares^42^. This is likely because violence can cause feelings of anxiety, stress, and fear, which can interfere with the ability to relax and fall asleep. Furthermore, violence can disrupt normal sleep patterns and routines, leading to difficulty sleeping. It is important for adolescents who have experienced violence to seek support and seek help if they are experiencing sleep problems as a result. In some cases, therapy or medication may be helpful in managing sleep difficulties related to violence^43^. Furthermore, psychological interventions, namely mindfulness meditation, also indicated positive results in improving sleep quality^44,45^.

According to the logistic regression analysis of the present study, parental involvement can indicate that it may serve as a protective element to minimize psychological distress (such as loneliness, worrying, and poor sleep quality). Strong and positive relationships among family members can provide a sense of support and security. It can also be an important protective factor against violence and bullying^46^. On the other hand, unhealthy relationships within a family can contribute to an environment where violence and bullying are more likely to occur. There are many ways that families can work to promote healthy relationships and reduce violence and bullying. Some strategies might include: (1) setting clear boundaries and expectations for behavior^47^; (2) encouraging open communication and listening to one another^48^; (3) resolving conflicts peacefully and with respect^49^; (4) seeking support from a therapist or counselor if needed^50^; (5) educating family members about the impact of violence and bullying^51^; and (6) the importance of being kind and respectful to one another^52^. It is important to note that addressing violence and bullying within a family can be challenging, and it may be necessary to seek outside help in order to create lasting change. Seeking support from therapists, psychologists, or community organizations specialized in these issues can help identify family-related stressors and perceptions of violence^53^.

Having close friends was also found to be a protective factor in this current study in both sexes. Previous evidence suggests that social relationships are able to prevent loneliness and sleep disturbance. In other words, feeling satisfied with one’s social interactions is strongly correlated with reduced loneliness in all age groups^54^. Furthermore, perceived support from peers is associated with improved sleep quality. There is evidence to suggest that perceived social support, including support from peers, can be associated with improved sleep quality^55^. For example, a study found that adolescents who reported higher levels of social support from their peers had better sleep quality and were less likely to experience sleep problems^56^. The way to address loneliness was suggested to educate people on how to build and maintain positive relationships, such as friendships, and provide the necessary support for these relationships to thrive^57^.

The findings of this research suggest that violence and bullying victimization (inside and outside schools) have a negative and notable influence on the mental health and well-being of adolescents. It is important to understand the root causes and relevant context of these issues in order to effectively address them and promote the well-being of adolescents. Adolescents are particularly vulnerable to the effects of violence and are bullied due to their developmental stage and the central role that social relationships play in their lives. These experiences can have long-term consequences on mental health, including an increased risk of anxiety, depression, and suicidality. Given the serious and lasting effects of violence and bullying on the mental health of adolescents, it is essential to implement strategies to prevent these issues and to provide support and intervention for those who have experienced traumatic experiences. It is well established that addressing violence and bullying is necessary for promoting the overall well-being of adolescents. Effective prevention and intervention strategies may include educating adolescents about healthy relationships and communication, providing resources and support for those who have experienced violence or are bullied, and promoting a culture of respect for others and inclusion within schools and communities. By addressing these issues, we can work towards creating a safer and more supportive environment for adolescents to thrive.

In addition, protective factors, specifically, parental involvement can be an important factor in minimizing psychological distress, including loneliness, worrying, and sleep problems, in children and adolescents. Research has shown that children and adolescents who have supportive and involved parents tend to have better mental health outcomes^58,59^, and are less likely to experience psychological distress. Parental involvement can take many forms, including participating in activities with children, providing emotional support and encouragement, setting clear expectations and boundaries, and being available to talk and listen to children. By providing a supportive and nurturing environment, parents can help reduce the likelihood that children experience psychological distress and can also help mitigate the negative impact of stressors when they occur.

## Study limitations

The GSHS measure is based on self-reports and only assessed from adolescents attending school. Therefore, there could be recall bias and the prevalence of psychological distress may be different in adolescents not attending school. Furthermore, cross-sectional survey data do not infer causality between psychosocial factors, protective factors, and psychological distress. In this study psychological distress was only assessed with a 2-item screener, and future research may want to include full psychological distress scales.

## Conclusions

More than one in five school-going adolescents in Bolivia had psychological distress. Psychosocial factors, including physical attacks, physical fighting, and bullying victimisation (including in and outside schools and cyberbullied) are one of the key factors that influence psychological distress, especially worry-induced sleep disturbance and loneliness. On the other hand, a healthy relationship with parents and having close friends was protective against psychological distress. According to the evidence presented above, it is the responsibility of all those (parents, teachers, school staff, and so on) who are looking after children and adolescents to understand the factors that can cause their psychological problems, and also know how to support and care for them. Children and adolescents’ caregivers may be able to have better aspects of a supportive and nurturing environment to address the problem and may assist in the positive development of children and adolescents in terms of health, well-being, and a good shape of developmental stages by understanding the root causes of psychological distress, such as loneliness, worry, and sleep difficulties.

## Data Availability

The data on which this paper is based are available at the World Health Organization NCD Microdata Repository, at https://extranet.who.int/ncdsmicrodata/index.php/catalog/881/get-microdata.

## References

[1] Cole A, Bond C, Qualter P, Maes M (2021). A systematic review of the development and psychometric properties of loneliness measures for children and adolescents. Int. J. Environ. Res. Public Health.

[2] Twenge JM, Haidt J, Blake AB, McAllister C, Lemon H, Le Roy A (2021). Worldwide increases in adolescent loneliness. J. Adolesc..

[3] Moeller RW, Seehuus M (2019). Loneliness as a mediator for college students’ social skills and experiences of depression and anxiety. J. Adolesc..

[4] Beutel ME, Klein EM, Brähler E, Reiner I, Jünger C, Michal M (2017). Loneliness in the general population: prevalence, determinants and relations to mental health. BMC Psychiatry.

[5] Escobar DFSS, de Jesus TF, Noll PRES, Noll M (2020). Family and school context: Effects on the mental health of Brazilian students. Int. J. Environ. Res. Public Health.

[6] Tian S, Zhang TY, Miao YM, Pan CW (2021). Psychological distress and parental involvement among adolescents in 67 low-income and middle-income countries: A population-based study. J. Affect. Disord..

[7] Pengpid S, Peltzer K (2021). Prevalence and associated factors of loneliness among national samples of in-school adolescents in four caribbean countries. Psychol Rep..

[8] Pengpid S, Peltzer K (2021). Prevalence and associated factors of psychological distress among a national sample of in-school adolescents in Liberia. J. Psychol. Afr..

[9] Pengpid S, Peltzer K (2020). Prevalence and associated factors of psychological distress among a national sample of in-school adolescents in Morocco. BMC Psychiatry.

[10] Marthoenis, Dahlia, Nassimbwa J (2022). Prevalence and factors associated with loneliness among Indonesian female adolescents: a cross-sectional study. BMC Womens Health.

[11] Putra IGNE, Pradnyani PE, Putra GW, Astiti NLEP, Derayanti NW, Artini NNA (2022). Gender differences in social environmental factors of psychological distress among Indonesian adolescents: Findings from the 2015 global school-based student health survey. J. Biosoc. Sci..

[12] Pengpid S, Peltzer K (2021). Psychological distress among a national sample of school adolescents in Tonga: Prevalence and correlates. Int. J. Disabil. Hum. Develop..

[13] Li Y, Cong X, Chen S, Li Y (2021). Relationships of coping styles and psychological distress among patients with insomnia disorder. BMC Psychiatry.

[14] Nunes ML, Bruni O (2015). Insomnia in childhood and adolescence: clinical aspects, diagnosis, and therapeutic approach. J. Pediatr..

[15] Beck NI, Arif I, Paumier MF, Jacobsen KH (2016). Adolescent injuries in argentina, bolivia, chile, and uruguay: Results from the 2012–2013 global school-based student health survey (GSHS). Injury.

[16] Oldfield J, Humphrey N, Hebron J (2016). The role of parental and peer attachment relationships and school connectedness in predicting adolescent mental health outcomes. Child Adolesc. Mental Health.

[17] Moen ØL, Hall-Lord ML (2019). Adolescents’ mental health, help seeking and service use and parents’ perception of family functioning. Nordic J. Nurs. Res..

[18] Delgado E, Serna C, Martínez I, Cruise E (2022). Parental attachment and peer relationships in adolescence: A systematic review. Int. J. Environ. Res. Public Health.

[19] Serna C, Martínez I (2019). Parental involvement as a protective factor in school adjustment among retained and promoted secondary students. Sustainability..

[20] Mackova J, Veselska ZD, Geckova AM, Jansen DEMC, van Dijk JP, Reijneveld SA (2022). The role of parents in the care for adolescents suffering from emotional and behavioral problems. Front Psychol..

[21] Hasumi T, Ahsan F, Couper CM, Aguayo JL, Jacobsen KH (2012). Parental involvement and mental well-being of Indian adolescents. Indian Pediatr..

[22] Nguyen HTL, Nakamura K, Seino K, Al-Sobaihi S (2019). Impact of parent–adolescent bonding on school bullying and mental health in Vietnamese cultural setting: Evidence from the global school-based health survey. BMC Psychol..

[23] Semahegn A, Dessie Y, Assefa N, Canavan CR, Berhane Y, Fawzi WW (2021). Physical fighting among adolescents in eastern Ethiopia: a cross-sectional study. BMC Public Health.

[24] Lee LK, Chen PCY, Lee KK, Kaur J (2007). Violence-related behaviours among Malaysian adolescents: a cross sectional survey among secondary school students in Negeri Sembilan. Ann. Acad. Med. Singap..

[25] Shaikh MA, Abio A, Celedonia KL, Lowery WM (2019). Physical fighting among school-attending adolescents in Pakistan: Associated factors and contextual influences. Int. J. Environ. Res. Public Health.

[26] Omer M, Shaikh MA, Stiller M, Lowery WM (2020). Physical fighting among school-attending adolescents in El Salvador: An examination of the 2013 global school-based health survey. Int. J. Environ. Res. Public Health.

[27] Gabrielli S, Rizzi S, Carbone S, Piras EM (2021). School interventions for bullying-cyberbullying prevention in adolescents: Insights from the UPRIGHT and CREEP projects. Int. J. Environ. Res. Public Health.

[28] Craig W, Harel-Fisch Y, Fogel-Grinvald H, Dostaler S, Hetland J, Simons-Morton B (2009). A cross-national profile of bullying and victimization among adolescents in 40 countries. Int. J. Public Health..

[29] Lee JJ, Kim JH, Kim BN (2021). Effects of school bullying prevention camp on the adolescent perpetrators of school violence. J. Korean Acad. Child Adolesc. Psychiatry.

[30] Marthoenis M, Schouler-Ocak M (2022). Investigating the prevalence of psychological distress and its associated factors among Indonesian adolescents: A cross-sectional study. Child. Adolesc. Soc. Work J..

[31] Neupane T, Pandey AR, Bista B, Chalise B (2020). Correlates of bullying victimization among school adolescents in Nepal: Findings from 2015 global school-based student health survey Nepal. PLoS ONE.

[32] Cañas E, Estévez E, León-Moreno C, Musitu G (2020). Loneliness, family communication, and school adjustment in a sample of cybervictimized adolescents. Int. J. Environ. Res. Public Health.

[33] Garaigordobil M, Mollo-Torrico JP, Machimbarrena JM, Páez D (2020). Cyberaggression in adolescents of Bolivia: Connection with psychopathological symptoms, adaptive and predictor variables. Int J Environ Res Public Health..

[34] Ministry of Health. Bolivia (Plurinational State of 2018)—Global school-based student health survey 2018. (2021) [cited 2023 Jul 13]. https://extranet.who.int/ncdsmicrodata/index.php/catalog/881

[35] Brener ND, Collins JL, Kann L, Warren CW, Williams BI (1995). Reliability of the youth risk behavior survey questionnaire. Am. J. Epidemiol..

[36] Becker AE, Roberts AL, Perloe A, Bainivualiku A, Richards LK, Gilman SE (2010). Youth health-risk behavior assessment in Fiji: The reliability of Global School-based Student Health Survey content adapted for ethnic Fijian girls. Ethn. Health.

[37] Sharma B, Lee TH, Nam EW (2017). Loneliness, insomnia and suicidal behavior among school-going adolescents in western pacific island countries: role of violence and injury. Int. J. Environ. Res. Public Health..

[38] Pengpid S, Peltzer K (2022). Loneliness is associated with poor mental health, social-environmental factors, and health risk behaviours among national samples of in-school adolescents in four Caribbean countries. Psychol. Health Med..

[39] Povedano A, Cava MJ, Monreal MC, Varela R, Musitu G (2015). Victimization, loneliness, overt and relational violence at the school from a gender perspective. Int. J. Clin. Health Psychol..

[40] Donoghue C, Meltzer LJ (2018). Sleep it off: Bullying and sleep disturbances in adolescents. J. Adolesc..

[41] Kliewer W, Lepore SJ (2015). Exposure to violence, social cognitive processing, and sleep problems in urban adolescents. J. Youth Adolesc..

[42] Wolke D, Lereya ST (2015). Long-term effects of bullying. Arch. Dis. Child..

[43] World Health Organization. Medicines used in generalized anxiety and sleep disorders [Internet]. Pharmacological Treatment of Mental Disorders in Primary Health Care. Geneva, Switzerland: World Health Organization; (2009) [cited 2022 Dec 18]. https://www.ncbi.nlm.nih.gov/books/NBK143206/

[44] Black DS, O’Reilly GA, Olmstead R, Breen EC, Irwin MR (2015). Mindfulness meditation and improvement in sleep quality and daytime impairment among older adults with sleep disturbances: A randomized clinical trial. JAMA Intern. Med..

[45] Rusch HL, Rosario M, Levison LM, Olivera A, Livingston WS, Wu T (2019). The effect of mindfulness meditation on sleep quality: a systematic review and meta-analysis of randomized controlled trials. Ann. N. Y. Acad. Sci..

[46] Eşkisu M (2014). The relationship between bullying, family functions, perceived social support among high school students. Proc. Soc. Behav. Sci..

[47] Innis G. Boundaries and expectations are important parenting tools [Internet]. MSU Extension. (2012) [cited 2023 Jul 10]. https://www.canr.msu.edu/news/boundaries_and_expectations_are_important_parenting_tools

[48] Semeniuk YY, Brown RL, Riesch SK (2016). Analysis of the efficacy of an intervention to improve parent-adolescent problem solving. West J. Nurs. Res..

[49] Riesch SK, Gray J, Hoeffs M, Keenan T, Ertl T, Mathison K (2003). Conflict and conflict resolution: Parent and young teen perceptions. J Pediatr. Health Care..

[50] Baker-Ericzén MJ, Jenkins MM, Haine-Schlagel R (2013). Therapist, parent, and youth perspectives of treatment barriers to family-focused community outpatient mental health services. J Child Fam Stud..

[51] Gómez-Ortiz O, Apolinario C, Romera EM, Ortega-Ruiz R (2019). The role of family in bullying and cyberbullying involvement: examining a new typology of parental education management based on adolescents’ view of their parents. Soc. Sci..

[52] Otake K, Shimai S, Tanaka-Mataumi J, Otsui K, Fredrickson BL (2006). Happy people become happier through kindness: A counting kindnesses intervention. J Happiness Stud..

[53] Ware LM, Fortson BL, McNeil CB (2003). Parent-child interaction therapy: A promising intervention for abusive families. Behav. Anal. Today..

[54] Nicolaisen M, Thorsen K (2017). What are friends for? Friendships and loneliness over the lifespan-from 18 to 79 years. Int J Aging Hum Dev..

[55] Kent RG, Uchino BN, Cribbet MR, Bowen K, Smith TW (2015). Social relationships and sleep quality. Ann Behav Med..

[56] Stafford M, Bendayan R, Tymoszuk U, Kuh D (2017). Social support from the closest person and sleep quality in later life: Evidence from a British birth cohort study. J. Psychosom. Res..

[57] Hong JH, Yeh CS, Sandy LG, Fellows A, Martin DC, Shaeffer JA (2022). Friendship and loneliness: A prototype roadmap for health system action. Am J Prev Med..

[58] Butler N, Quigg Z, Bates R, Jones L, Ashworth E, Gowland S (2022). The contributing role of family, school, and peer supportive relationships in protecting the mental wellbeing of children and adolescents. Sch. Ment. Heal..

[59] Thomas PA, Liu H, Umberson D (2017). Family relationships and well-being. Innov. Aging..

